# LncRNA MIR17HG inhibits non-small cell lung cancer by upregulating miR-142-3p to downregulate Bach-1

**DOI:** 10.1186/s12890-020-1112-3

**Published:** 2020-03-30

**Authors:** Sen Wei, Jinghao Liu, Xin Li, Xingyu Liu

**Affiliations:** Department of Lung Cancer Surgery, General Hospital of Tianjin Medical University, No. 154 An’shan Road, Tianjin, 300052 China

**Keywords:** Non-small cell lung cancer, MIR17HG, miR-142-3p, Bach-1

## Abstract

**Background:**

This study aimed to investigate the role of MIR17HG in non-small cell lung cancer (NSCLC).

**Methods:**

Differential expression of MIR17HG in NSCLC was first detected by exploring the TCGA dataset. Expression levels of miR-142-3p in both NSCLC and non-tumor tissues were determined by qPCR. The effects of overexpressing MIR17HG on the methylation of miR-142 were assessed by MSP. The effects of overexpressing MIR17HG, miR-142-3p and Bach-1 on the invasion and migration of NSCLC cells were assessed by Trasnwell invasion or migration assay.

**Results:**

Analysis of TCGA dataset revealed slightly downregulated expression of MIR17HG in NSCLC. This downregulation was further confirmed by measuring the expression levels of MIR17HG in NSCLC and non-tumor tissues from NSCLC patients. MIR17HG was found to decrease the methylation of miR-142-3p, and overexpression of MIR17HG led to upregulated miR-142-3p. Moreover, overexpression of MIR17HG also led to downregulated Bach-1, the downstream target of miR-142-3p. Cell invasion and migration analysis showed that overexpression of MIR17HG and miR-142-3p led to inhibited cancer cell invasion and migration. In contrast, overexpression of Bach-1 played an opposite role and attenuated the effects of overexpressing MIR17HG and miR-142-3p.

**Conclusion:**

MIR17HG inhibits NSCLC by upregulating miR-142-3p to downregulate Bach-1.

**Trial registration:**

TJ-MU-2012-0148594, registered January 2, 2012

## Background

Lung cancer has been the most common type of malignancy that causes the highest mortality rate among all types of malignancies for decades [1]. In 2018, lung cancer affected 11.6% (2,093,876 cases) of all newly diagnosed cancer cases and caused 18.4% (1,761,007 cases) of all cancer deaths [2]. Avoidance of smoking and second-hand smoking can significantly reduce the risk of lung cancer [3]. However, to quit smoking, it requires intensive intervention for smokers’ life. More importantly, lung cancer also affects never-smokers [4]. The current survival of lung cancer patients is still poor, largely due to the lack of effective therapies for advanced lung cancer and the low rate of early diagnosis [5, 6].

Genetic factors play central roles in the pathogenesis of lung cancer [7]. The identification of genetic factors involved in this disease provided potential targets for the development of targeted therapies [8, 9]. Non-coding RNAs (ncRNAs) have no protein-coding capacity but affect protein synthesis by regulating gene expression [10]. For instance, long (> 200 nt) non-coding RNAs (lncRNAs) are usually spatially expressed and affect local gene expression [11]. However, the functions of most lncRNAs in cancer remain unclear. LncRNA miR-17-92a-1 cluster host gene (MIR17HG) has been reported to produce cancer-related miRNAs [12]. For instance, MIR17HG produces miRNA-19a/b to mediate the anti-neoplastic effects induced by grape seed procyanidin extract in the treatment of lung cancer [13]. MIR17HG was also reported to promote colorectal cancer by interacting with miR-17-5p [14]. In the present study, we observed downregulated expression pattern of MIR17HG in NSCLC by analyzing TCGA dataset. In addition, our preliminary deep sequencing data revealed positive correlation between the expression of MIR17HG and miR-142-3p, a tumor suppressive miRNA [15], across NSCLC tissue samples (data not shown). This study aimed to investigate the potential involvement of MIR17HG in non-small cell lung cancer (NSCLC), a major subtype of lung cancer, and explore its possible interactions with miR-142-3p.

## Materials and methods

### Patients and tissue samples

Paired non-tumor and NSCLC tissue samples were obtained from 60 NSCLC patients (41 males and 19 females, 42 to 68 years old, mean age 54.3 ± 5.8 years old). These patients were admitted by General Hospital of Tianjin Medical University between March 2012 and May 2014. This study passed the review board of the Ethics Committee of aforementioned hospital. All patients were diagnosed for the first time. No therapies for any clinical disorders were performed within 3 months before admission. Patients complicated with other clinical disorders were excluded from this study. Based on AJCC clinical staging system, the 60 patients included 14, 16 and 30 at clinical stage II, III, and IV, respectively. All patients were informed of the experimental design of this project and signed the informed consent.

### NSCLC cells and transfection

This study used human NSCLC cell lines H2170 and H2126 as the cell model of NSCLC. No mutations were observed in these two cell lines. Cells were obtained from ATCC (USA). Cells were cultured in cell culture medium composed of 90% RPMI-1640 medium and 10% FBS at 37 °C with 95% humidity and 5% CO_2_. Cells were harvested at confluence of 70–80% for transfections. MIR17HG and Bach-1 expression vectors were constructed using pcDNA3.1 vector as the backbone. Negative control (NC) miRNA and miR-142-3p mimic were synthesized by RIBOBIO (Guangzhou, China). Lipofectamine 2000 (Invitrogen, USA) was used to transfect 10 nM vector (NC group was empty pcDNA3.1 vector transfection) or 45 nM miRNA (NC group was NC miRNA transection). Untransfected cells were used as the control cells (C). The following experiments were conducted 24 h post-transfections.

### RNA extractions and qRT-PCR

Ribozol (Sigma-Aldrich) was used to perform all RNA extractions following the manufacture’s instructions. RNA samples were precipitated using 85% ethanol to harvest miRNAs. All RNA samples were digested by DNA eraser (Takara, USA). SSRT IV system (Thermo Fisher Scientific) was used to transcribe total RNAs into cDNAs. With cDNA samples as template, SYBR® Green Realtime PCR Master Mix (Toyobo, Japan) was used to prepare all qPCR mixtures. GAPDH was used as the endogenous control to measure the expession levels of MIR17HG and Bach-1. All-in-One™ miRNA qRT-PCR Detection Kit (GeneCopoeia) was used to measure the expression levels of mature miR-142-3p. All steps including polyadenylation, reverse transcriptions and qPCR assays were performed following the instructions from GeneCopoeia. U6 was used as the endogenous control. All qPCR assays were repeated 3 times and data normalizations were performed using 2^-ΔΔCq^ method.

### Methylation specific PCR (MSP)

Genomic DNAs were extracted from H2170 cells at 24 h post-transfection using the Wizard® Genomic DNA Purification Kit (Promega Corporation). EZ DNA Methylation-Gold™ Kit (ZYMO RESEARCH, USA) was used to convert DNA samples. DNA methylation was measured by performing PCR reactions using the Taq 2X Master Mix (NEB).

### Transwell assays

The effects of transfections on the invasion and migration of cells were evaluated by Transwell invasion and migration assays, respectively. Transwell insert (Corning) was used for cell migration assay. The upper chamber was coated with Matrigel for cell invasion assay. The upper chamber was filled with 500 μl non-serum cell suspension containing 10^5^ cells, while the lower chamber was filled with a mixture of 20% FBS and 80% aforementioned medium. Cells were cultivated under aforementioned conditions for 12 h, followed by staining of the lower membrane surface using 0.5% crystal violet (Sigma-Aldrich). The stained cells were observed under a light microscope.

### Western blot

RIPA solution (Sigma-Aldrich) was used for protein extractions. All proteins samples were incubated in boiling water for 10 min for denaturation. Electrophoresis was then performed using 12% SDS-PAGE gel. After that, protein samples were transferred to PVDF membranes, followed by blocking in PBS containing 5% non-fat milk. The blocked membranes were then incubated with primary antibodies of rabbit anti-GAPDH (ab9485, 1:1400, Abcam) and Bach-1 (ab7288, 1:1400, Abcam) primary antibodies. IgG-HRP secondary antibody (ab6721, 1:1400, Abcam) was then used to incubate with the membranes. Signals were detected using ECL (Sigma-Aldrich, USA). Image J v1.48 software was used to normalize the expression levels of Bach-1 to GAPDH.

### Statistical analysis

Mean values of 3 independent biological replicates were calculated. Comparisons between two groups were performed using paired t test. Comparisons among multiple groups were performed using one-way ANOVA and Tukey test. Correlation analysis was performed by linear regression. *P* < 0.05 was statistically significant.

## Results

### MIR17HG was downregulated in NSCLC

**Fig. 1:**
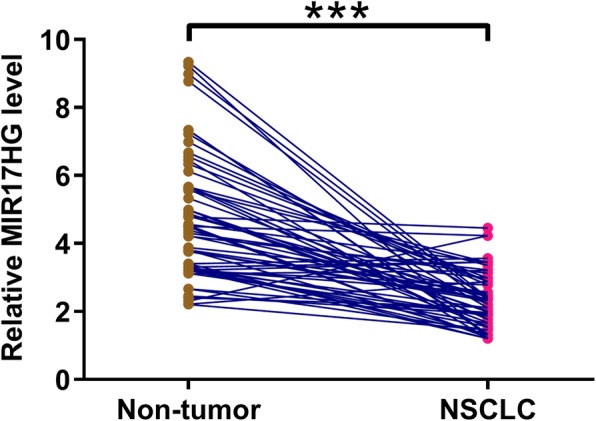
MIR17HG was downregulated in NSCLC. Expression levels of MIR17HG in both NSCLC and non-tumor tissues of the 60 NSCLC patients included in this study were measured by performing qPCR. PCR reactions were repeated 3 times and mean values were presented. ***, *p* < 0.001

Differential expression of MIR17HG in NSCLC was first detected by exploring the TCGA dataset. It was observed that MIR17HG was downregulated in both adenocarcinoma (0.4 vs. 0.54) and squamous cell carcinoma (0.76 vs. 0.88), which are two major subtypes of NSCLC. To further confirm this, the expression levels of MIR17HG in both NSCLC and non-tumor tissues of the 60 NSCLC patients were measured by qPCR. Paired t test analysis showed that the expression levels of MIR17HG were significantly lower in NSCLC tissues than that in non-tumor tissues (Fig. 1, *p* < 0.001).

### MiR-142-3p was positively correlated with MIR17HG

**Fig. 2:**
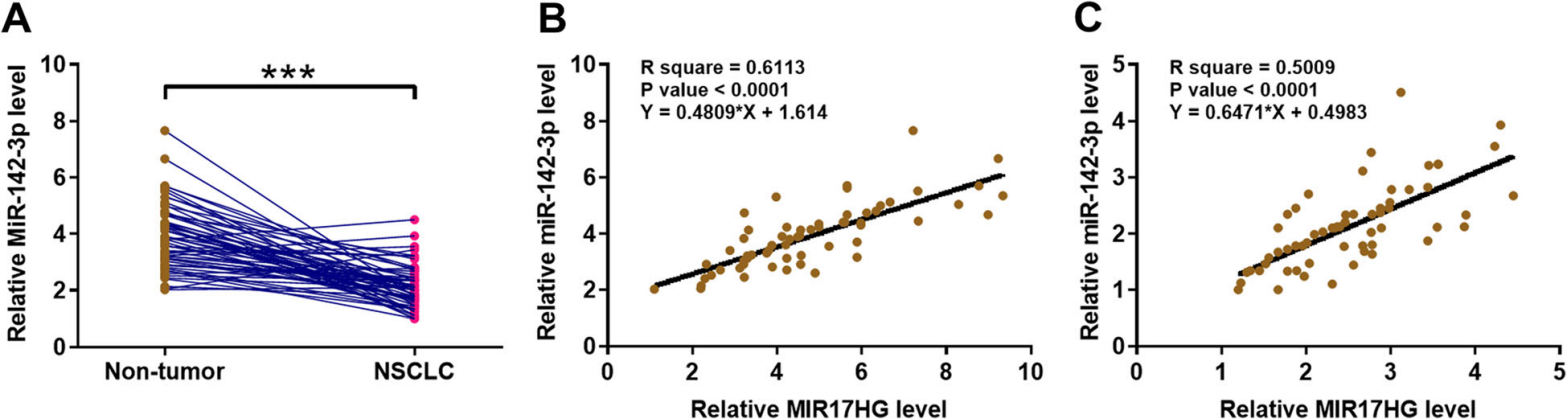
MiR-142-3p was positively correlated with MIR17HG. Expression of miR-142-3p in both NSCLC and non-tumor tissues was determined by qPCR (**a**). Correlations between miR-142-3p and MIR17HG across both NSCNC (**b**) and non-tumor (**c**) tissue samples were analyzed by linear regression. PCR reactions were repeated 3 times and mean values were presented.***, *p* < 0.001

Expression of MiR-142-3p in both NSCLC and non-tumor tissues were also determined by qPCR. It was observed that the expression levels of miR-142-3p were also significantly lower in NSCLC tissues than that in non-tumor tissues (Fig. 2 a, *p* < 0.001). Correlation analysis showed that the expression of miR-142-3p and MIR17HG were positively correlated across both NSCNC (Fig. 2 b) and non-tumor (Fig. 2 c) tissue samples.

### Overexpression of MIR17HG decreased methylation of miR-142 and downregulated Bach-1

**Fig. 3:**
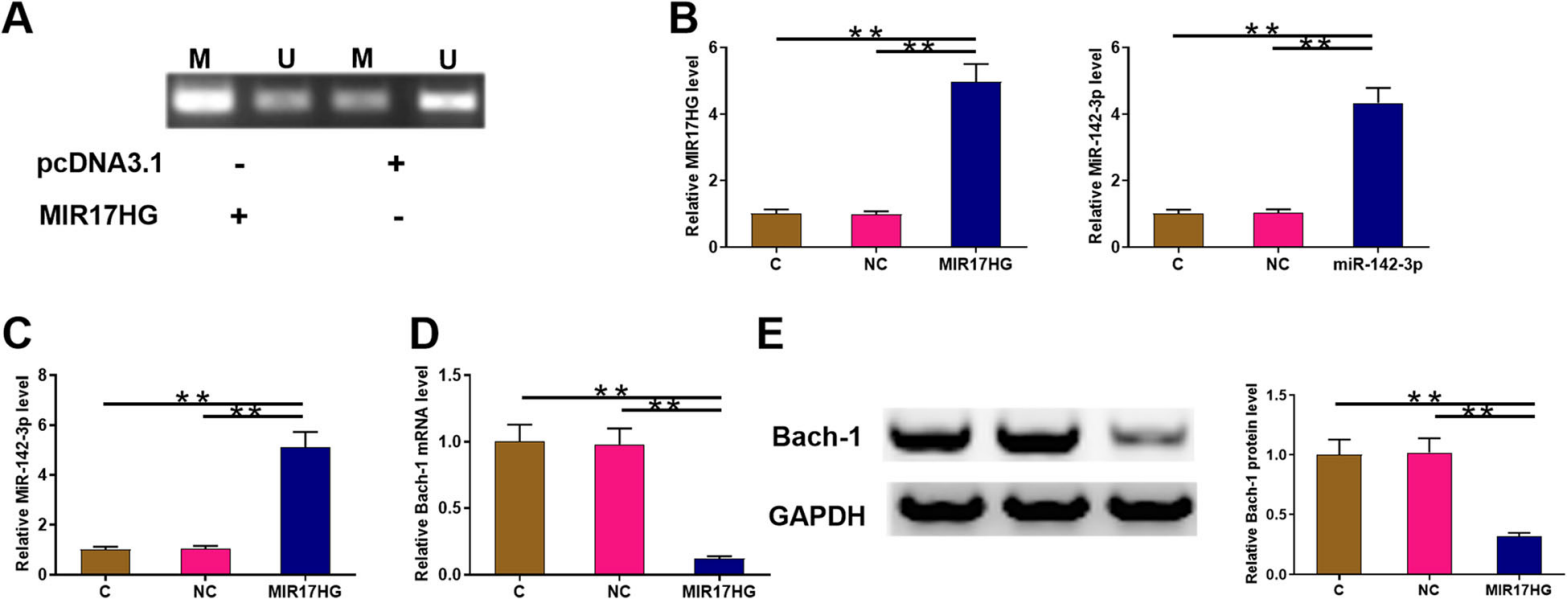
Overexpression of MIR17HG decreased miR-142 methylation and downregulated Bach-1. The effects of overexpressing MIR17HG on miR-142 gene methylation were analyzed by MSP (**a**). To further analyze the relationship between MIR17HG and miR-142-3p, cells were transfected with MIR17HG vector or miR-142-3p mimic. Overexpression of MIR17HG and miR-142-3p was confirmed by qPCR at 24 h post-transfection (**b**). The effects of overexpressing MIR17HG on miR-142-3p was analyzed by qPCR (**c**). Effects of overexpressing MIR17HG on the expression of Bach-1 at both mRNA and protein levels were analyzed by qPCR and western blot, respectively (**d**). All experiments were repeated 3 times and mean values were presented. M, methylation; U, unmethylation; *, *p* < 0.05

The effects of overexpressing MIR17HG on the methylation of miR-142 were assessed by MSP. Compared to cells transfected with pcDNA3.1 vector, cells transfected with MIR17HG expression vector showed obviously reduced methylation of miR-142 gene (Fig. 3 a). To further investigate the relationship between MIR17HG and miR-142-3p, cells were transfected with MIR17HG vector or miR-142-3p mimic. Overexpression of MIR17HG and miR-142-3p were confirmed by qPCR at 24 h post-transfection (Fig. 3 b, *p* < 0.05). It showed that overexpression of MIR17HG lead to upregulation of miR-142-3p (Fig. 3 c, *p* < 0.05). Overexpression of MIR17HG led to downregulation of Bach-1, which is a target of miR-142-3p, at mRNA level (Fig. 3 e, *p* < 0.05). Representative western blot results were presented (Fig. 3 e, left, *p* < 0.05), and the results showed that overexpression of MIR17HG led to downregulation of Bach-1 at protein level as well (Fig. 3 e, right, *p* < 0.05).

### MIR17HG inhibited invasion and migration of cancer cells through miR-142-3p and Bach-1

**Fig. 4:**
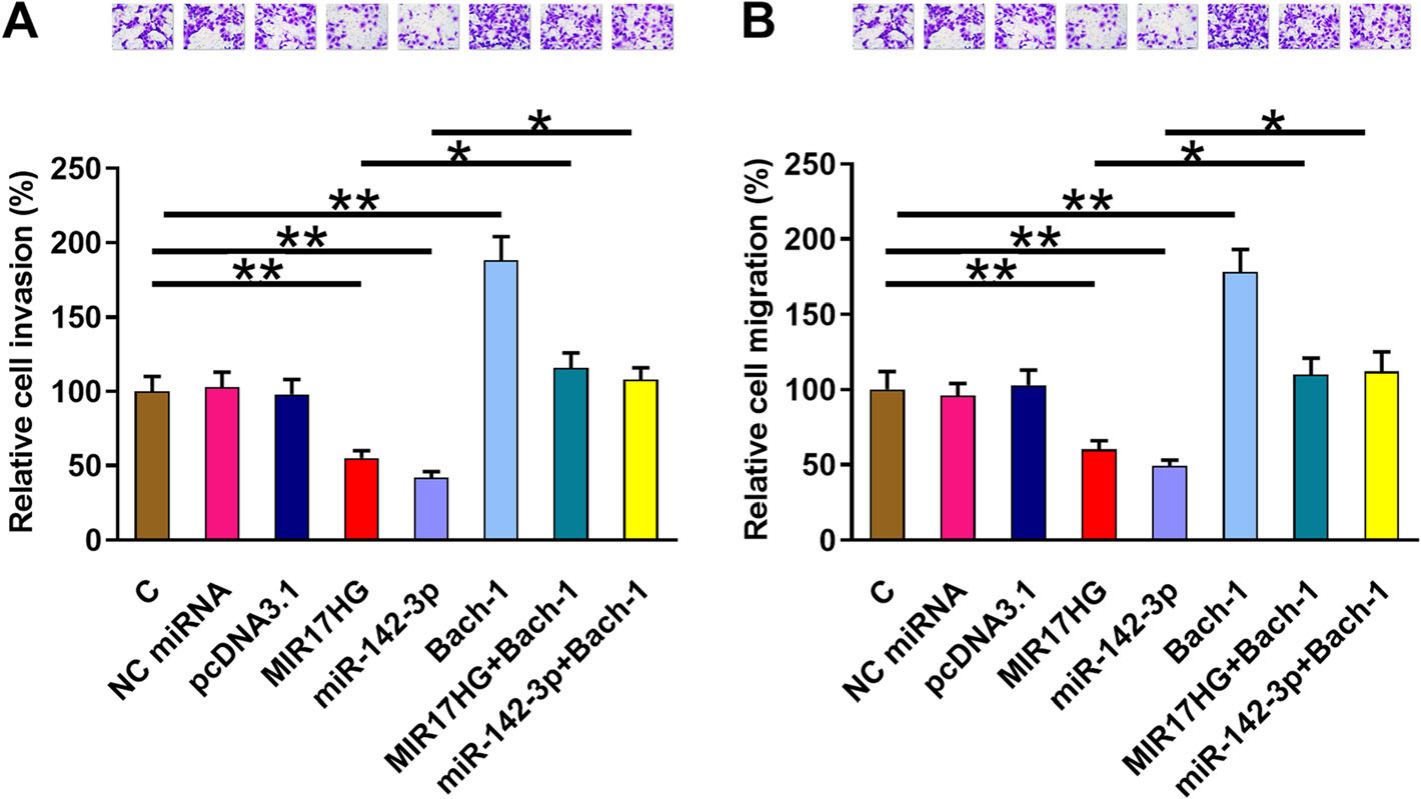
MIR17HG inhibited invasion and migration of H2170 cells through miR-142-3p and Bach-1. The effects of overexpressing MIR17HG, miR-142-3p and Bach-1 on the invasion (**a**) and migration (**b**) of H2170 cells were assessed by Trasnwell invasion or migration assay. All experiments were repeated 3 times and mean values were presented. *, *p* < 0.05

The effects of overexpressing MIR17HG, miR-142-3p and Bach-1 on the invasion (Fig. 4 a) and migration (Fig. 4 b) of NSCLC cells were assessed by Trasnwell invasion or migration assay. Compared to the Control (C) group or cells transfected with pcDNA3.1 vector or NC miRNA, overexpression of MIR17HG and miR-142-3p led to inhibited cancer cell invasion and migration. In contrast, overexpression of Bach-1 played an opposite role and attenuated the effects of overexpressing MIR17HG and miR-142-3p (*p* < 0.05). To further confirm the effects of MIR17HG, miR-142-3p and Bach-1 in regulating NSCLC cell invasion and migration, Transwell invasion (Supplemental Fig. 1A) and migration (Supplemental Fig. 1A) assays were repeated using another cell line H2126, and similar results were obtained.

## Discussion

This study mainly investigated the roles of MIR17HG in NSCLC. We observed that MIR17HG was downregulated in NSCLC and may upregulate the expression miR-142-3p by reducing the methylation of this gene.

Expression patterns of MIR17HG in different types of cancer have not been well studied. It is reported that MIR17HG is the host of multiple oncogenic miRNAs that involved in the progression of cell death [12]. However, downregulation of MIR17HG was observed in many types of cancer after analyzing the TCGA dataset, such as acute myeloid leukemia (6.91 vs. 32.83), ovary cancer (0.88 vs. 1.77) and pancreas adenocarcinoma (0.84 vs. 1.64), which suggests the tumor suppressive roles of MIR17HG in these types of cancer. We also observed the slightly lower levels of MIR17HG in NSCLC tissues compared to that in non-tumor tissues by analyzing TCGA dataset. Significantly reduced expression levels of MIR17HG were observed in NSCLC tissues compared to that in non-tumor tissues in the 60 NSCLC patients included in this study. This is possibly due to the different populations included in these two datasets. Moreover, our study observed reduced invasion and migration rates of NCSLC cells after the overexpression of MIR17HG. Therefore, MIR17HG may play tumor suppressive roles in NSCLC.

It is commonly observed that tumor suppressive miRNAs are frequently methylated in cancers [15–17]. For instance, miR-145 is regulated by DNA methylation in prostate cancer [15]. MicroRNA-34b/c is downregulated in gastric cancer through methylation-related pathways [16]. MiR-142-3p is a well-established tumor suppressive miRNA and has regulatory roles in multiple cell behaviors, such as migration and invasion. A recent study reported that miR-142 is hypermethylated in liver cancer [17]. Our study also observed the methylation of miR-142 in NSCLC cells. It is known that lncRNAs can regulate the methylation of miRNA genes [18]. In this study we observed that overexpression of MIR17HG reduced the methylation of miR-142-3p gene to upregulate its expression, and this expression regulation was involved in the invasion of migration of NSCLC cells. However, the mechanisms of methylation regulation of miR-142-3p by MIR17HG are still unclear.

It is known that Bact-1 in cancer biology is involved in the regulation of heme oxidation and oxidative stress. It is also linked to cancer invasion and metastasis [19]. Therefore, MIR17HG may directly regulate Bach-1 through miR-142-3p to regulate NSCLC cell invasion and migration. It is worth noting that we also tried to measure the expression levels of Bach-1 in paired tissues. However, Bach-1 is below detectable level in many non-tumor samples. This is possibly due to the low expression levels of Bach-1 in some patients. In our future studies, we will increase the sensitivity of qPCR to solve this problem.

## Conclusion

In conclusion, MIR17HG was downregulated in NSCLC and may upregulate miR-142-3p through methylation pathway to promote its expression, thereby downregulating Bach-1 and suppress cancer cell invasion and migration.

## Supplementary information

**Additional file 1: **Supplemental Fig. 1 MIR17HG inhibited invasion and migration of H2126 cells through miR-142-3p and Bach-1. The effects of overexpressing MIR17HG, miR-142-3p and Bach-1 on the invasion (A) and migration (B) of H2126 cells were assessed by Trasnwell invasion or migration assay. All experiments were repeated 3 times and mean values were presented. *, *p* < 0.05.

## Data Availability

The analyzed data sets generated during the study are available from the corresponding author on reasonable request.

## Abbreviations

ncRNAs: Non-coding RNAs
lncRNAs: Long (> 200 nt) non-coding RNAs
NSCLC: Non-small cell lung cancer
