# Supplementary figures and images for: LncRNA MIR17HG inhibits non-small cell lung cancer by upregulating miR-142-3p to downregulate Bach-1
Source: BMC Pulm Med. 2020 Mar 30;20:78. doi: 10.1186/s12890-020-1112-3 (PMC7104535; doi:10.1186/s12890-020-1112-3)

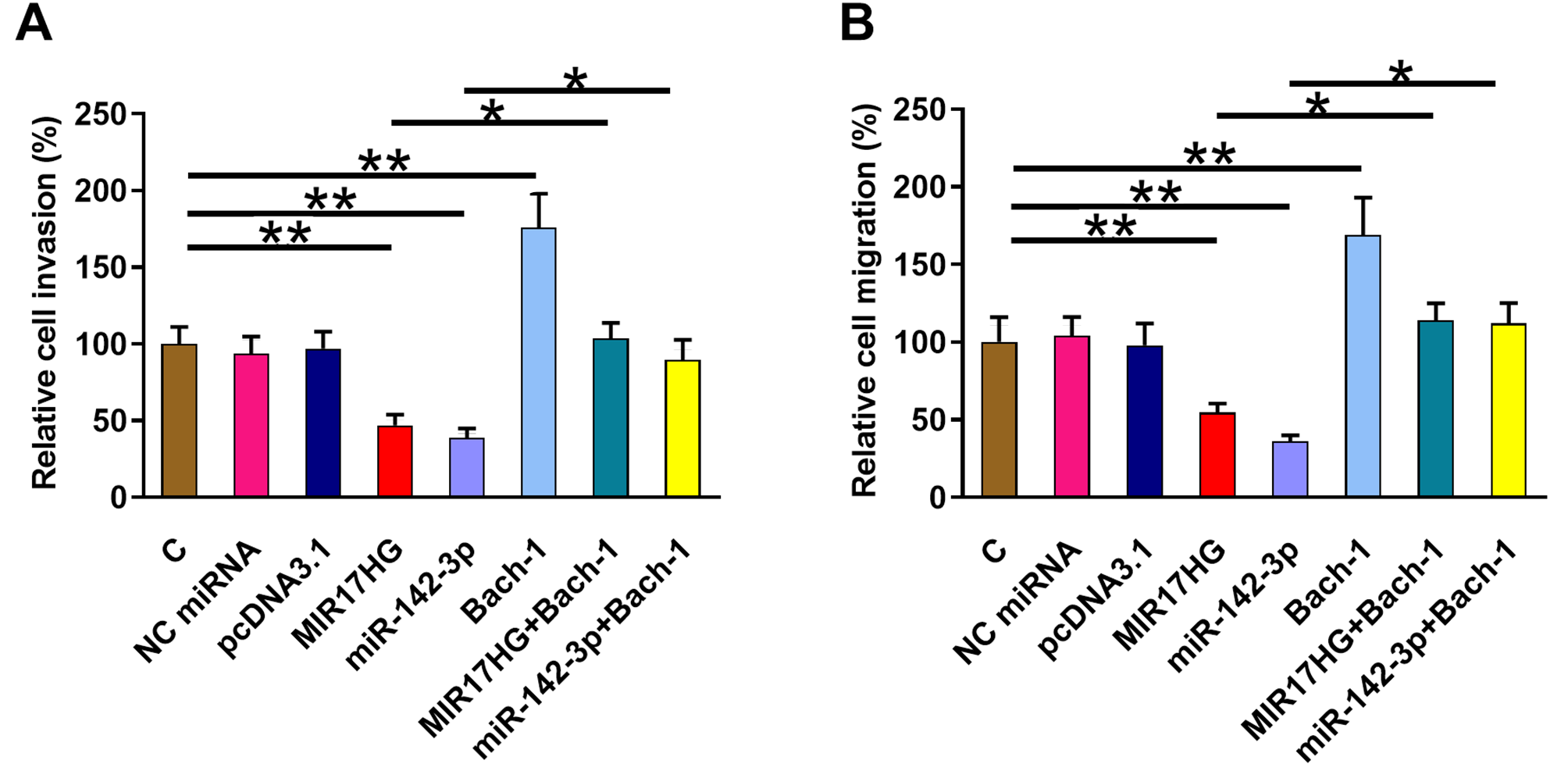

Supplement: Supplementary file 1 — Additional file 1: Supplemental Fig. 1 MIR17HG inhibited invasion and migration of H2126 cells through miR-142-3p and Bach-1. The effects of overexpressing MIR17HG, miR-142-3p and Bach-1 on the invasion (A) and migration (B) of H2126 cells were assessed by Trasnwell invasion or migration assay. All experiments were repeated 3 times and mean values were presented. *, p < 0.05. [file 12890_2020_1112_MOESM1_ESM.tif]
